# Characterization of mechanical and radiation isocenter on an MR‐guided radiotherapy (MRgRT) Linac

**DOI:** 10.1002/acm2.14111

**Published:** 2023-08-03

**Authors:** Nema Bassiri, John E. Bayouth, Kathryn E. Mittauer

**Affiliations:** ^1^ Department of Radiation Oncology Miami Cancer Institute Baptist Health South Florida Miami Florida USA; ^2^ Herbert Wertheim College of Medicine Florida International University Miami Florida USA; ^3^ Department of Radiation Medicine Oregon Health and Science University Portland Oregon USA

**Keywords:** isocenter, mechanical, MLC, MRgRT, MR‐guidance, MR‐RT QA

## Abstract

**Background and Purpose:**

In the emerging paradigm of stereotactic radiosurgery being proposed for MR‐guided radiotherapy (MRgRT), assessment of mechanical geometric accuracy is critical for the implementation of stereotactic delivery. We benchmarked the mechanical accuracy of an MR Linac system that lacks an onboard detector/array. Our mechanical tests utilize a half beam block (HBB) geometry that takes advantage of the sensitivity of a partially occluded detector.

**Materials and Methods:**

Mechanical tests benchmarked the couch, MLC, and gantry geometric accuracy for an MR‐Linac system. An HBB technique was used to irradiate an ionization chamber profiler (ICP) array with partial occlusion of individual detectors for characterization of MLC skew, beam divergence displacement, and RT isocenter localization. The sensitivity of the partially occluded detector's ICP‐X (detector width) and ICP‐Y (detector length) was characterized by displacing the detector relative to radiation isocenter by 0.2 mm increments, introduced through couch motion. The accuracy of the HBB ICP technique was verified with a starshot using radiochromic film, and the reproducibility was verified on a conventional C‐arm Linac and compared to Winston‐Lutz.

**Results:**

The sensitivity of the HBB technique as quantified through the dose difference normalized to open field as a function of displacement from RT isocenter was 6.4%/mm and 13.0%/mm for the ICP‐X and ICP‐Y orientation, respectively, due to the oblong detector orientation. Couch positional accuracy and sag was within ±0.1 mm. Maximum MLC positional displacement was 0.7 mm with mean MLC skew at 0.07°. The maximum beam divergence displacement was 0.03 mm. The gantry angle was within 0.1°. Independent verification of the RT isocenter localization procedure produced repeatable results.

**Conclusion:**

This work serves for characterizing the mechanical and geometric radiation accuracy for the foundation of an MR‐guided stereotactic radiosurgery program, as demonstrated with high sensitivity and independent validation.

## 1 INTRODUCTION

The accuracy of the radiation delivery is dependent on the mechanical and geometric components of a Linac system. Specifically, radiation isocenter represents a sphere in space where the central axis (CAX) of all radiation beams intersects. Traditional C‐arm Linacs use gantry, collimator, and couch angles to maximize their ability to deliver radiation. The CAX of any beam emitted from any given combination of these mechanical properties is designed to intersect at the radiation isocenter. In comparison, O‐shaped Linacs, that is, TomoTherapy (Accuray Inc., Madison WI), Halcyon, (Varian Medical Systems, Palo Alto, CA), MRIdian (ViewRay Inc., Denver, CO), do not have a rotating collimator or couch, and instead only rotate a Linac in a circle about a fixed axis of rotation, that is, the mechanical isocenter. The mechanical isocenter should coincide with the radiation isocenter.

Historically, the starshot technique has been used to determine the radiation isocenter in O‐shaped and C‐arm Linacs. This technique measures the radiation isocenter using the intersection of beam centers across a variety of gantry angles.^1^ Each individual beam CAX is determined across the profile's full width at half max (FWHM), and the isocenter is determined by the confluence of each ray with regards to its CAX. The starshot has traditionally been performed using film,^1^, ^2^, ^3^ electronic portal imaging device (EPID)^4^, ^5^ or other detectors.^6^, ^7^ Specifically, groups have applied starshot techniques to verify MR‐RT isocenter using in‐house cameras and/or phantoms to detect Cherenkov radiation or optical emission.^6^, ^7^ While these proposed solutions can quantify the radiation isocenter size and location, they require expensive, non‐commercial detectors and phantoms.

Recently, ViewRay released a stereotactic brain package (BrainTx™) as part of its A3i system, which motivates evaluating the MRIdian for stereotactic radiosurgery/radiotherapy (SRS/SRT) applications. The MRIdian system lacks an onboard detector or EPID. As such, a practical method to precisely quantify and localize radiation isocenter is needed beyond current techniques. The Winston‐Lutz test is the gold standard for measuring isocenter with SRS level precision (i.e., <1 mm).^8^ Lutz et al. developed a technique to quantify the displacement of a BB projected with radiation at any collimator, gantry, and couch angle.^9^ The BB's position is measured for each field which determines the location of isocenter.

An approach for localizing RT isocenter on MRIdian has been previously described by Latifi et al.^15^ in which the radiation isocenter is found using two pieces of radiochromic film and a proprietary cylindrical phantom. The first film must be precisely cut and placed in the transverse orientation (International Electrotechnical Commission (IEC)‐X and IEC‐Z axis), and the second film is wrapped around the phantom (IEC‐Y axis). Both films are marked at the laser crosshair before the phantom is translated into the center of the bore. A radiation starshot technique is performed, and the film is analyzed using software‐based analysis. This technique is limited due to the precision of marking the film, the small circumference of the phantom limiting the FWHM precision, and tedious setup/readout of the film. Furthermore, an iterative method of irradiating and scanning individual film can be quite time consuming and cumbersome when initially determining the location of isocenter.

In the emerging paradigm of stereotactic radiosurgery being proposed for MRIdian, assessment of mechanical geometric accuracy is critical for the implementation of stereotactic delivery. Currently there is no comprehensive reporting in the literature benchmarking the accuracy of the critical mechanical components of MRIdian radiation delivery (i.e., MLC skew, RT isocenter localization, beam divergence displacement, couch/gantry position). In this work, we propose a novel technique that utilizes a half beam block geometry which partially occludes a detector to precisely define radiation isocenter and mechanical precision of the ViewRay MRIdian. Our method uses an ionization chamber profiler array and takes advantage of the sensitivity gained from a partially occluded detector for a Linac system that lacks an onboard detector/array.

## 2 METHODS

### 2.1 Systems

#### 2.1.1 ViewRay MRIdian Linac

The MRIdian system and its multi‐leaf collimator (i.e., RayZR™ MLC), which has been previously described,^10^ has a 90 cm SAD and a 6 MV flattening filter free linear accelerator. The MLC is a double‐stacked, double‐focused, jawless design such that the upper bank (MLC1) and lower bank (MLC2) are offset by half a leaf width or 4.15 mm at isocenter. The MLCs travel in the same direction (IEC‐X at G0) for both banks. The x‐ray beam's central axis (CAX) is centered between leaf pair 17 and 18 for MLC1 and at the center of leaf pair 18 for MLC2. Each movable leaf is capable of full overtravel and interdigitation.

#### 2.1.2 ICP array

This study utilized an MR‐compatible ionization chamber profiler (ICP) array (IC PROFILER™‐MR, Sun Nuclear Inc., Melbourne, FL).^11^, ^12^ The ICP is a 32 × 32 cm^2^ detector array composed of 251 parallel plate chambers. There are 65 and 63 detectors (active volume 0.046 cm^3^ and 14.4 pC/cGy sensitivity) with 5.0 mm center to center spacing along ICP‐Y and ICP‐X axis, respectively, when placed flat on the treatment couch. The width, length, and height of ICP‐X and ICP‐Y parallel plate is 2.86, 7.3, and 3.3 mm. The ICP‐X and ICP‐Y detector spacing is 5 mm. The influence of the Lorentz force from the magnetic field on the ICP has been previously described.^12^

### 2.2 Mechanical assessment

#### 2.2.1 Overview

**FIGURE 1:**
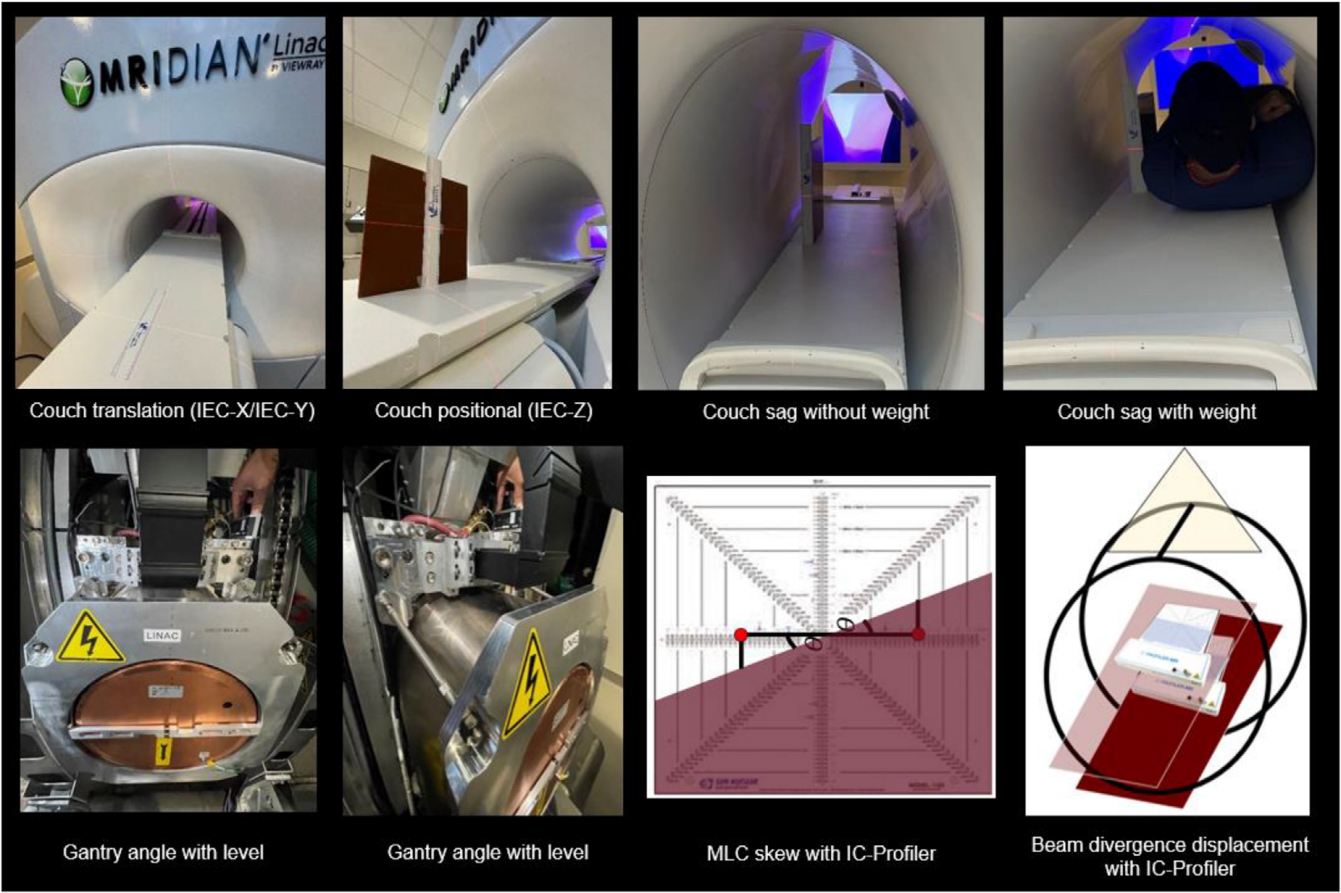
Overview of mechanical procedures to quantify for couch positional accuracy, couch sag, gantry angle accuracy, MLC skew rotation, and beam divergence displacement.

Mechanical tests benchmarked the couch, MLC, and gantry geometric accuracy. The mechanical positional accuracy also serves as a foundation to perform the proposed RT isocenter procedure. Figure 1 displays methods used to quantify mechanical accuracy of the couch positional accuracy, couch sag, gantry angle, MLC skew, and beam divergence displacement. These mechanical components are not only coupled, but also are used in the precision of finding RT isocenter (i.e., shifting couch small increments to localize RT isocenter throughout opposed gantry angles and across MLC leaf travel). The mechanical accuracy was assessed per the guidelines from TG 142 and TG 198.

#### 2.2.2 Couch sag and positional accuracy

The couch translational accuracy was performed per the recommendations outlined in TG 198. A ruler (Figure 1) was aligned to the lasers at the virtual isocenter (−155 cm in IEC‐Y from machine isocenter). Known shifts of 0.5 mm, 1 mm, 5 mm, and 100 mm were applied across longitudinal and vertical couch directions. The applied lateral couch shifts were 0.5 mm, 1 mm, 5 mm, and 50 mm due to lateral couch displacement limitations. For vertical couch shifts, a ruler was taped on solid water, as shown in Figure 1. The respective shifted couch positions were verified by visual inspection of the ruler.

Couch mechanical sag was performed by placing a weight at machine isocenter within the bore. A weight of 150 pounds was placed on the couch, and the IEC‐Z position of the laser relative to its initial position without weight was quantified.

#### 2.2.3 MLC positional accuracy

An MLC picket fence assessed the accuracy of individual MLC leaves. The picket fence was performed with radiochromic film (Gafchromic EBT3, Ashland) positioned at isocenter for each cardinal gantry angle. Each bank (MLC1 and MLC2) was measured independently across all four gantry positions. Measurements were performed with film positioned between solid water of 5 cm for buildup and backscatter. The film was analyzed in RIT software version 6.8.64 (Radiological Imaging Technology, Inc., Colorado Springs, CO) to measure the absolute position of eight distinct junctions across MLC leaf travel direction.

#### 2.2.4 MLC skew

The MLC skew compared to the gantry axis of rotation was quantified using the ICP across opposing gantry angles at G0 and G180. For this technique, a half beam block was used, and the line profile across the detector was measured. If the detector is skewed, opposing line profiles, as measured by the ICP, will display the same directionality of angularity between G0 and G180. However, if the MLC banks are skewed, the line profiles will display opposing profiles of angularity. Note for this assessment of MLC skew, the ICP was manually rotated to remove detector rotation error. To this end, we iteratively rotated the ICP until the normalized off‐axis dose difference at +10 cm and −10 cm measured similar dose at G0.

The angle of MLC skew was calculated based on percentage differences between the detector dose at off‐axis positions with a half beam block across the ICP‐Y direction (leaf side) compared to an open field measurement at the same off‐axis detector position. The dose difference was calculated as a positional difference, as later described in our RT isocenter localization procedure, per Equation (2). The angle of rotation of the MLC (Figure 1) was quantified by calculating the arctangent of the off‐axis detector position (i.e., ± 5 cm and ± 10 cm) from CAX for gantry angles on the same plane (i.e., G0/G180).

#### 2.2.5 Linac alignment with gantry

Gantry angle was verified, per the recommended test from TG 198. A level was placed on the surface of the gantry ring that the Linac is the mounted to, as shown in Figure 1. The gantry was rotated until the level was plumb. The gantry angle was readout and compared to its known position.

#### 2.2.6 Beam divergence displacement

The displacement of the Linac beam divergence was quantified as a function of couch vertical motion. The ICP central detector was balanced in the IEC‐Y coordinate at G0 using HBBs created using the X2 MLC bank with respective open Y1 and Y2 orientations. The couch was positioned at its maximum and minimum vertical limits, and a set of HBBs and open field measurements were acquired. The HBBs were normalized to the open field CAX detector dose at each couch height. Then the displacement was quantified by converting the relative CAX dose differences to displacement using Equations (1) and (2). The beam divergence was quantified as the difference in the calculated displacement of the beam's CAX between the limits of the couch positions.

### 2.3 RT isocenter procedure overview

#### 2.3.1 Gantry orientation

**FIGURE 2:**
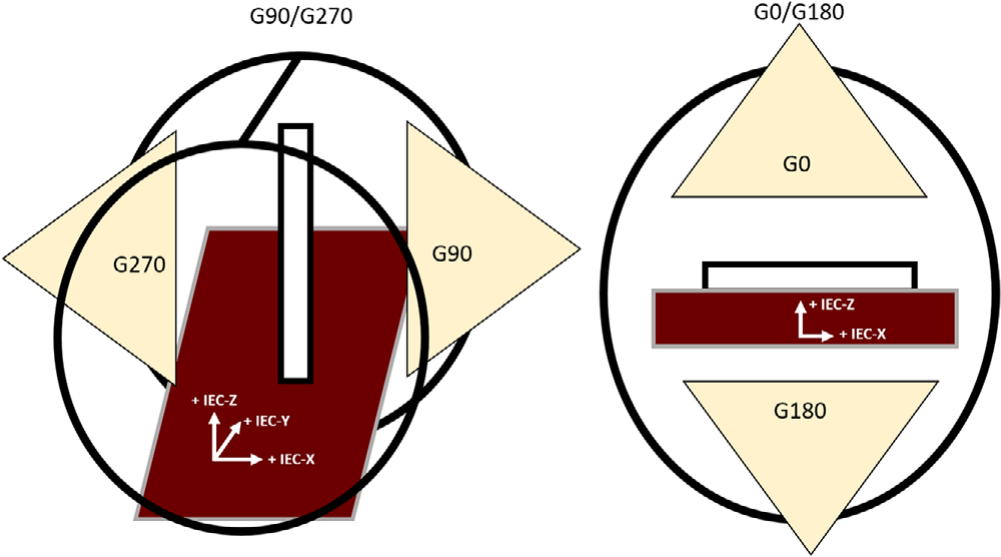
ICP orientations for G90/G270 IEC‐Y/IEC‐Z (left) and G0 IEC‐X/IEC‐Y (right) when placed in the bore, relative to its respective IEC coordinates. Note both G0 and G180 is used for initial detector alignment in the IEC‐X/IEC‐Y configuration. The beams associated with the ICP measurements have been labelled according to their respective cardinal angles, G0/G90/G180/G270.

**FIGURE 3:**
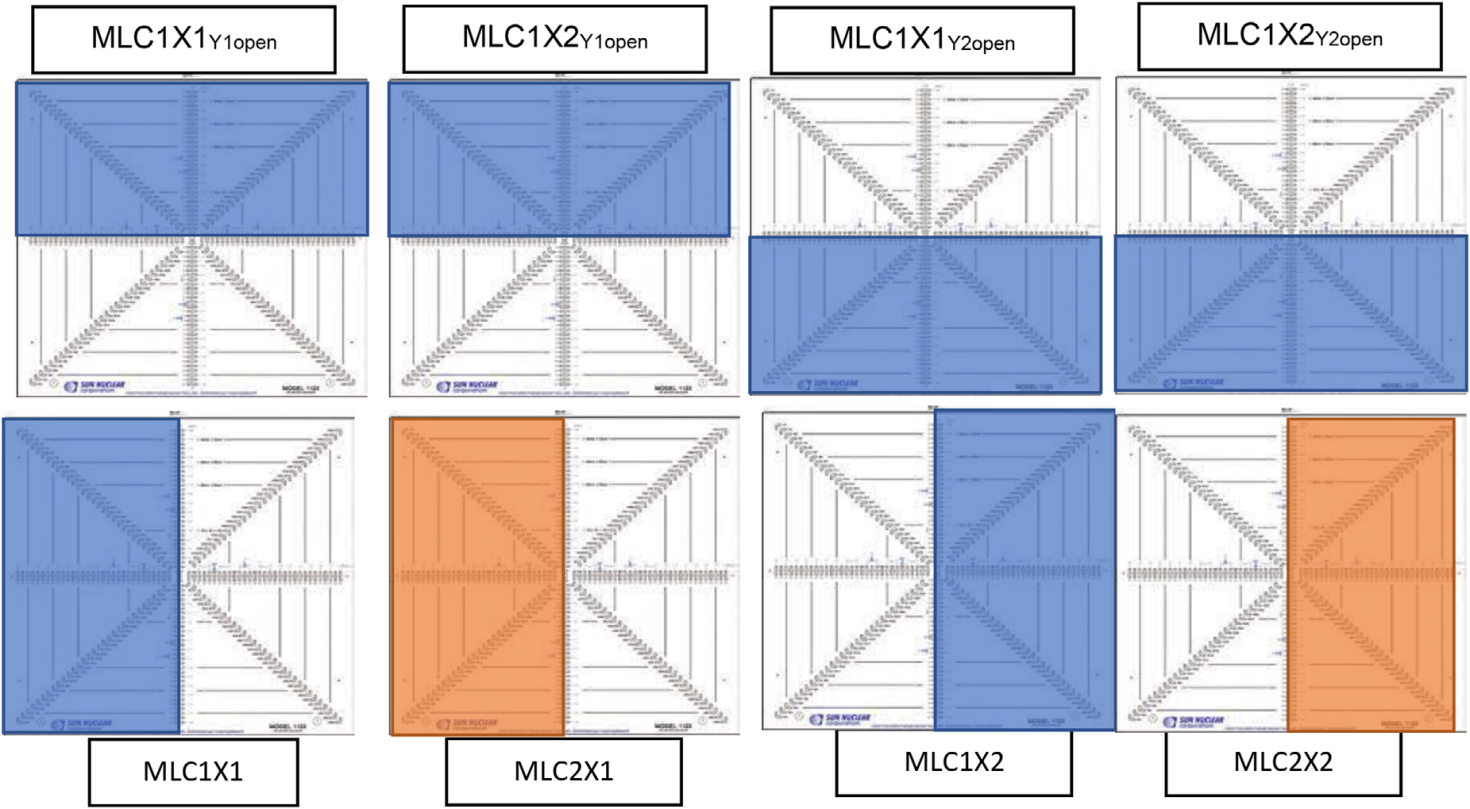
Eight MLC HBBs (MLC1X1_Y1open_, MLC1X2_Y1open_, MLC1X1_Y2open_, MLC1X2_Y2open_, MLC1X1, MLC1X2, MLC2X1, MLC2X2) per gantry cardinal angle were used to measure dose to the central detector. The solid‐colored blocks (orange and blue) describe the MLC creating a half beam block (HBB). The orange and blue HBBs represent the closing of the upper (MLC1) and lower (MLC2) MLC stack, respectively. The X describes which side of the MLC‐X bank (i.e., X1 vs. X2) is used to create the field. Open fields are denoted by the exposed ICP.

Radiation isocenter was evaluated across the cardinal angles using a half beam block (HBB) technique. The RT isocenter localization was performed for IEC‐X/IEC‐Y orientation at G0 and for IEC‐Y/IEC‐Z orientation at G90/G270. Figure 2 displays the ICP orientations for localizing RT isocenter in the IEC‐X/IEC‐Y (Figure 2, right) and IEC‐Y/IEC‐Z (Figure 2, left). Note while G180 was used for initial detector alignment in the IEC‐X/IEC‐Y configuration (Figure 2, right), HBB measurements (Figure 3) were not acquired at G180 due to couch attenuation (∼15%) and non‐uniformity of radiation transmission through the couch guide.

#### 2.3.2 HBB fields

For the three cardinal angles (G0/G90/G270), eight distinct half beam block measurements at central axis were taken, as shown in Figure 3. The eight HBB orientations are listed in Table 1. MLC1 and MLC2 represent the upper and lower MLC, respectively. X1 and X2 denotes which bank (i.e., X1 or X2) is blocking or defining the field edge. Furthermore, MLC#X#_Y#open_ denotes either Y1 or Y2 being exposed in the HBB either the IEC‐Y (G0) or IEC‐Y (G90/G270) axis (Figure 3, top row).

**TABLE 1:** IC Profiler versus IEC coordinate system as function of gantry angle for half beam block (HBB) measurements.

Gantry orientation	IEC coordinate	ICP coordinate	HBB Fields
G0	IEC‐X	ICP‐X	MLC1X1, MLC1X2, MLC2X1, MLC2X2
G0	IEC‐Y	ICP‐Y	MLC1X1_Y1open_, MLC1X1_Y2open_, MLC1X2_Y1open_, MLC1X2_Y2open_
G90/G270	IEC‐Z	ICP‐Y	MLC1X1, MLC1X2, MLC2X1, MLC2X2
G90/G270	IEC‐Y	ICP‐X	MLC1X1_Y1open_, MLC1X1_Y2open_, MLC1X2_Y1open_, MLC1X2_Y2open_

#### 2.3.3 ICP orientation

The ICP was positioned flat on the couch in the IEC‐X/IEC‐Y orientation for the G0 measurement. For the G90/G270 measurement in the IEC‐Y/IEC‐Z orientation, the ICP was positioned upside down while rotated 90 degrees. For both configurations, the detector was placed in the assumed plane of isocenter, which is 90 cm source to detector distance.

The measurement either utilized the X or Y detector row on the ICP, and will be referred to as, ICP‐X and ICP‐Y orientation, respectively. The ICP‐X orientation defines the radiation field using the MLC leaf end and splits the parallel plate chamber along the lateral axis. The ICP‐Y orientation defines the radiation field using the MLC leaf side and splits the parallel plate chamber along the longitudinal axis. Note that for ICP‐Y (i.e., IEC‐Y at G0 and G90/270) HBB measurements utilized only MLC1, as displayed in Table 1. MLC1's two central leaves (i.e., Leaf 17 and Leaf 18) split the isocenter in half. Furthermore, MLC2 fully blocks the isocenter, and a 50% occlusion of isocenter is not possible. Note for this study, an index bar was used to align the ICP array orthogonal on the couch to minimize detector skew and to create a reproducible setup for benchmarking institutional baseline data.

**TABLE 2:** MLC skew characterized with IEC‐Y half beam block at detector positions ± 50 mm ± 100 mm.

	detector dose (cGy)				%DD		converted to position (mm)		arctan [converted position/detector position] (degrees)	
Detector position (mm)	Open field G0	MLC1X2Y1_open_ G0	Open field G180	MLC1X2Y1_open_ G180	G0	G180	G0	G180	G0	G180
−100	71.11	36.05	56.37	30.05	51%	53%	0.09	−0.12	−0.05	0.07
−50	88.25	45.13	72.28	38.43	51%	53%	0.05	−0.11	−0.06	0.13
50	87.95	44.12	73.36	38.31	50%	52%	0.13	−0.03	0.15	−0.04
100	70.86	35.75	58.87	30.62	50%	52%	0.11	−0.02	0.06	−0.01

### 2.4 Procedure

#### 2.4.1 Detector alignment

For initial detector alignment, we calculated the displacement of the ICP relative to isocenter. The ICP was leveled and aligned to the room lasers. Open fields of 4.98, 9.98, 15.78, and 19.92 cm^2^ were delivered to the ICP across the four cardinal gantry angles at respective detector orientation (Figure 2). Four field sizes were used to average across the uncertainty of the interpolated position of beam center as reported by the ICP.

The isocenter displacement, relative to the CAX, was calculated by the ICP software and was taken as the initial couch shift for detector alignment. This technique was repeated for each respective detector orientation, gantry positions at G0/G180 and G90/G270, prior to measuring HBB fields.

#### 2.4.2 RT isocenter assessment

The RT isocenter was assessed through HBB across CAX normalized to an open field measurement on an ICP. As such, we used a similar approach to our previously published technique^10^ and Simon et al.^13^ in which the detector response of the partial occluded detector of the HBB field is normalized to an open field with the MLC retracted. This enabled normalization of output due to differences in attenuation when irradiating the posterior portion of the ICP, that is, G270 compared to G90. An open field was taken as 24.70 × 27.04 cm^2^, in accordance with the HBB technique previously described.^10^

#### 2.4.3 G90/G270: IEC‐Z

To find isocenter in the IEC‐Z, four HBB measurements were taken across each gantry angle of G90/G270. As previously detailed in Figure 3 and Table 1, four HBB measurements (i.e., MLC1X1, MLC1X2, MLC2X1, MLC2X2) are used. These designed HBBs incorporated differences in the upper and lower MLC banks (i.e., MLC1 and MLC2) as well as the right and left banks (X1 and X2). Of note these HBBs designs are defined by the leaf end and therefore subjected to MLC positional accuracy of a particular leaf. The detector response was averaged across the central ten detectors on the ICP array to minimize the impact of random leaf calibration errors.

**FIGURE 4:**
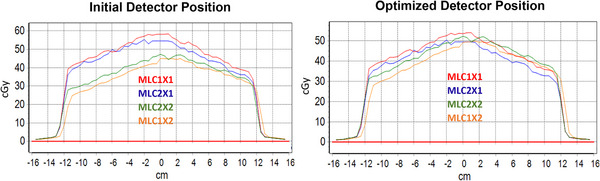
Left: An example of the profiles when the detector position is offset from isocenter, that is, overexposure (blue and red profiles) and underexposure (green and orange profiles). Right: optimized detector position.

The four MLC shapes were balanced (i.e., equal detector response) across the two cardinal angles G90 and G270. The detector board was shifted iteratively between measurements until a balanced detector output was measured across the four MLC configurations. The couch coordinate which yielded the tightest grouping among all four measurement configurations was taken to be the optimized detector position at IEC‐Z. Figure 4 (left) shows an example of the profiles when the detector position is offset from isocenter, that is, overexposure (blue and red profiles) and underexposure (green and orange profiles). Figure 4 (right) shows an optimized detector position. Once the optimal IEC‐Z position was determined, the ICP was moved 155 cm out of the bore and the lasers were adjusted to this detector position.

#### 2.4.4 G90/G270: IEC‐Y

The isocenter at IEC‐Y was taken across three cardinal angles of G0, G90, and G270. First, isocenter was measured at G90/G270 in the ICP‐X orientation then at G0 in the ICP‐Y orientation.

Four HBB measurements were taken across each gantry angle of G90/G270 using MLC1. As shown in Figure 3 and Table 1, four HBB measurements (i.e., MLC1X1_Y1open_, MLC1X1_Y2open_, MLC1X2_Y1open_, MLC1X2_Y2open_) which split isocenter using each of the four central leaves (i.e., MLC1X1 Leaf 17, MLC1X1 Leaf 18, MLC1X2 Leaf 17, and MLC1X2 Leaf 18) are used. These designed HBBs incorporated differences in MLC track travel and leaf construction (i.e., X1 Leaf 17, X2 Leaf 17, X1 Leaf 18, and X2 Leaf 18). Hence, we are quantifying our RT isocenter across these four MLC quadrants.

The couch was shifted iteratively if the HBB CAX measured dose between opposing IEC‐Y HBBs (i.e., Y1 open vs. Y2 open) differed by more than 8%. The 8% was based on an inability to get a perfect balance due to imperfections in leaf and MLC track construction. This threshold was chosen as ±4% dose difference which represented a sub‐millimeter agreement in RT isocenter location. The detector was positioned to balance the radiation exposure among the four MLC configurations until the global minimum was found. Once isocenter was determined, the IEC‐Y lasers were then positioned to this initial detector configuration from G90/G270.

#### 2.4.5 G0: IEC‐Y

IEC‐Y isocenter was remeasured using G0 in the ICP‐Y configuration. The MLC corresponding shapes, described in Figure 3: MLC1X1_Y1open_, MLC1X1_Y2open_, MLC1X2_Y1open_, MLC1X2_Y2open_, were used. The final IEC‐Y isocenter was determined by splitting the final couch coordinates between G0 and G90/G270. The lasers were then adjusted to the IEC‐Y isocenter position.

#### 2.4.6 G0: IEC‐X

Lastly, the isocenter at IEC‐X was taken at G0 for ICP‐X configuration. Similar to IEC‐Z G90/G270 orientation, the detector response was averaged across the central ten detectors on the ICP array to average out uncertainty of the MLC calibration of any one leaf.

The four MLC shapes, as described in Figure 3 (i.e., MLC1X1, MLC1X2, MLC2X1, MLC2X2) were balanced iteratively. As previously described, the detector board was shifted iteratively until a balanced output was determined and provided the tightest grouping across all four MLC configurations. The lasers were adjusted to the IEC‐X isocenter.

### 2.5 Characterization of accuracy & sensitivity of technique

#### 2.5.1 Assessment of sensitivity

The sensitivity of our partially occluded detector technique was evaluated. Since the parallel plate detector of the ICP array is oblong, the sensitivity differences due to detector geometry was assessed. To this end, the ICP‐Y (longitudinal) and ICP‐X (lateral) axis of the ICP's central detector was individually characterized as a function of couch/detector displacement relative to the RT isocenter. All measurements taken were at G0 using the ICP‐X and ICP‐Y orientation, as shown in Figure 2.

The lateral sensitivity measurement was determined using the ICP‐X orientation with MLC1X1 and MLC1X2. Similarly, the longitudinal sensitivity across the ICP‐Y orientation and MLC1X2_Y1open_ and MLC1X2_Y2open_ was found. For both sets of measurements, the ICP was placed at RT isocenter. The HBB measurements were taken in 0.2 mm increments as the couch/detector ranged from −1.4 mm to +1.4 mm relative to isocenter. The percentage dose difference of the central detector for the HBB versus the 24.70 × 27.04 cm^2^ open field was reported.

#### 2.5.2 Validation of technique: Starshot

Our ICP HBB method to establish isocenter was independently verified using a starshot (IEC‐X/IEC‐Z) and slit (IEC‐Y) measurement on radiochromic film. Prior to radiation, an MR‐compatible BB was positioned at the center of the film. A pinprick through the film was precisely placed at the center of the BB position. The BB was aligned to the respective laser position, established from the HBB measurements, with the film perpendicular to the couch for the IEC‐X/IEC‐Z, and parallel to the couch for IEC‐Y. A ten‐spoke starshot plan was delivered with a five‐field fixed beam of 1 × 24.07 cm^2^ with 400 MU per gantry angle. For IEC‐Y verification, a slit measurement of 27.04 × 1.66 cm^2^ with 1000 MU was performed.

The displacement of the lasers to the RT isocenter was quantified between the pinprick on each film and its respective central axis of radiation. The film was analyzed in RIT software. The magnitude of displacement between film RT CAX and the laser position was compared to the position of the isocenter previously established using our ICP HBB technique.

#### 2.5.3 Validation of technique: C‐arm

Our RT isocenter localization technique was repeated on a conventional C‐arm Linac to verify its reproducibility. The ICP was aligned to the lasers, and the RT isocenter was found by shifting the couch iteratively until a balanced HBB CAX was measured. The recorded shifts were reported relative to the initial laser alignment. A Winston‐Lutz test (1 × 1 cm^2^ at cardinal angles) was then performed to quantify the displacement of lasers to RT isocenter. The root mean squared (RMS) displacement was compared between the Winston‐Lutz test and the ICP RT localization technique for G0 (IEC‐X and IEC‐Y) and G270 (IEC‐Y and IEC‐Z).

## 3 RESULTS

### 3.1 Mechanical assessment

The mechanical assessment was evaluated per TG 198 and TG 142 tolerances. The couch translational accuracy proved the necessary precision for shifting the MRIdian's couch. The greatest recorded displacement from known location was measured to be 0.1 mm when shifting the couch +5 mm and +50 mm in the IEC‐X direction. All other couch translations had no difference between the introduced shift and the final couch position across the three directions. The couch sag demonstrated that adding a weight of 150 pounds does not affect the position of the couch.

MLC positional accuracy was quantified across ± 5 cm IEC‐Y and 20 cm across leaf travel direction (IEC‐X). This sampling region was selected based on the area of interest needed to assess MLC accuracy for the RT isocenter localization procedure (i.e., central ten detectors used in HBB technique). The average absolute displacement measured for MLC1 and MLC2 was 0.22 ± 0.16 mm and 0.25 ± 0.16 mm with maximum displacement of 0.65 mm and 0.68 mm, respectively.

The MLC skew, as measured at detector positions ± 50 mm and ± 100 mm using the IEC‐Y HBB technique, is shown in Table 2. Measured dose to the CAX detector using HBBs, which are normalized to open fields, is shown in Table 2 (columns 2−5). The converted position (columns 8−9) utilized Equation (2) to quantify the displacement from dose difference. The tangent function (columns 10−11) was used to determine the skew angle as a function of the detector position (column 1) and the calculated displacement (columns 8−9). The average absolute MLC skew was found to be 0.08 and 0.06 degrees for G0 and G180, respectively.

The displacement of beam divergence, assessed as a function of vertical couch position, is shown in Table 3. The detector dose across IEC‐Y HBB (columns 2−4), when the couch was positioned at its maximum and minimum limits, was normalized to its respective open field (columns 5−6). The percentage dose relative to CAX was converted to displacement (columns 7−8) using Equations (1) and (2) for each couch position. The difference in displacement between the couch vertical limits, quantified by the respective IEC‐Y HBB (columns 7−8), was then taken as the beam divergence displacement (columns 9−10). The difference in beam diverge displacement was −0.02 and −0.03 mm for the open Y1 and Y2 fields, respectively.

**TABLE 3:** Beam divergence displacement characterized with IEC‐Y half beam block with couch vertical motion.

	detector dose (cGy)			%DD_CAX_		%DD_CAX_ converted to displacement (mm)		Beam divergence displacement (Δmm)	
Couch vertical motion (cm)	Open field G0	MLC1X2Y1_open_ G0	MLC1X2Y2_open_ G0	Y1 open	Y2 open	Y1 open	Y2 open	Y1 open	Y2 open
0	106.86	60.51	56.74	57%	53%	−0.39	−0.04	−0.02	−0.03
−19.9	71.53	40.31	38.26	56%	53%	−0.37	−0.01		

The mechanical gantry angle accuracy was verified within 0.1 degrees at G90 as measured by a level. In summary, all mechanical results were well within the 1 mm/1 degree tolerance recommended by TG 142, and the level of precision needed for the RT isocenter localization procedure.

### 3.2 Sensitivity characterization

**FIGURE 5:**
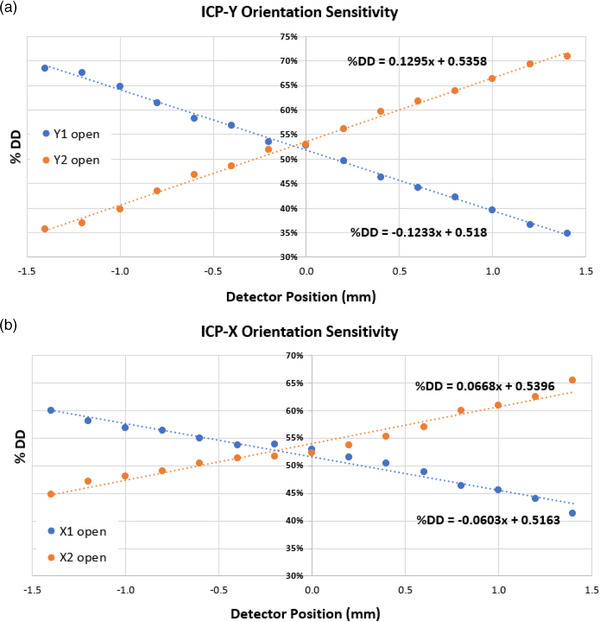
Characterization of the sensitivity of percentage dose difference of central detector (% DD_CAX_) for half beam block (HBB) across IC Profiler (ICP)‐Y (A) and ICP‐X (B) orientations as a function of detector displacement from RT isocenter.

The sensitivity of HBB along the ICP‐X and ICP‐Y orientations as a function of detector displacement from RT isocenter (∆_RTisocenter position_ [mm]) and percentage dose difference of central detector (%DD_CAX_), relative to the open field measurement (24.70 × 27.04 cm^2^) taken at isocenter, is displayed in Figure 5. Note for this characterization in ICP‐X configuration only the central detector dose difference was reported for simplicity rather than across the central ten detectors, since the MLC positional consistency between CAX and adjacent leaves was previously benchmarked. The ICP‐X orientation demonstrated less sensitivity for the measured dose with respect to detector position translated from RT isocenter. The relationship between detector response to RT isocenter positional change was found to be 13%/mm for ICP‐Y orientation and about 6.4%/mm for ICP‐X orientation [Figure 5, Equations (1)–(4)), slope of linear fit]:

(1) $$\begin{array}{ccc} & & \%{\text{DD}}_{\text{CAX}}\left({\text{ICP-Y}}_{\text{Y1open}}\right)\\ & & \;=0.130(\Delta_{\text{RTisocenter position}}\;\text{[mm]})+0.536 \end{array}$$

(2) $$\begin{array}{ccc} & & \%{\text{DD}}_{\text{CAX}}\left({\text{ICP-Y}}_{\text{Y2open}}\right)\\ & & \;=-0.123(\Delta_{\text{RTisocenter position}}\;\text{[mm]})+0.518 \end{array}$$

(3) $$\begin{array}{ccc} & & \%{\text{DD}}_{\text{CAX}}\left({\text{ICP-X}}_{\text{X1open}}\right)\\ & & =\;0.067(\Delta_{\text{RTisocenter position}}\;\text{[mm]})+0.540 \end{array}$$

(4) $$\begin{array}{ccc} & & \%{\text{DD}}_{\text{CAX}}\left({\text{ICP-X}}_{\text{X2open}}\right)\\ & & \;=-0.060(\Delta_{\text{RTisocenter position}}\;\text{[mm]})+0.516 \end{array}$$

Equations (1)–(4) describe the percent detector difference as a function of detector position, relative to isocenter. Equations (1)–(2) and (3)–(4) describe how the detector response varies in the ICP‐Y (*R* ^2^ = 0.997) and ICP‐X orientations (*R* ^2^ > 0.968), respectively.

### 3.3 RT isocenter assessment

#### 3.3.1 Square field measurements

The initial detector alignment for square field sizes, 4.98, 9.96, 15.78, and 19.92 cm^2^ in the G0/G180 and G90/G270 orientations, are provided in Table 4. The preliminary shifts as quantified by ICP beam center were −0.43, 1.19, and −0.43 mm for IEC‐X, IEC‐Y, and IEC‐Z, respectively. Note that Table 4 uses the ICP coordinate system and the couch shifts are relative to IEC convention. The magnitude of these shifts was taken as the average of the absolute value of the pre‐adjustment CAX shifts. The sign determined the direction of the necessary couch translations, relative to the IEC coordinate system. The initial absolute couch coordinates were 0, 259.84, and −18.26 cm for IEC‐X, IEC‐Y, and IEC‐Z.

**TABLE 4:** Preadjusted detector shifts as quantified as the beam center from ionization chamber profiler (ICP) with open square fields to localize ICP for initial radiation isocenter measurements.

Pre‐adjustment—G0		
FS (cm^2^)	*Y* (cm)	*X* (cm)
9.96	−0.15	−0.03
15.78	−0.12	−0.05
4.98	−0.14	−0.03
19.92	−0.15	−0.06
Average	−0.140	−0.043

Pre‐adjustment—G90		
FS (cm^2^)	*Y* (cm)	*X* (cm)
9.96	−0.03	0.11
15.78	−0.04	0.09
4.98	−0.03	0.1
19.92	−0.04	0.12
Average	−0.035	0.105

Pre‐adjustment—G180		
FS (cm^2^)	*Y* (cm)	*X* (cm)
9.96	−0.12	−0.05
15.78	−0.11	−0.05
4.98	−0.12	−0.04
19.92	−0.14	−0.03
Average	−0.123	−0.043

Pre‐adjustment—G270		
FS (cm^2^)	*Y* (cm)	*X* (cm)
9.96	−0.06	0.11
15.78	−0.06	0.09
4.98	−0.05	0.11
19.92	−0.04	0.13
Average	−0.050	0.110

#### 3.3.2 HBB field measurements

**FIGURE 6:**
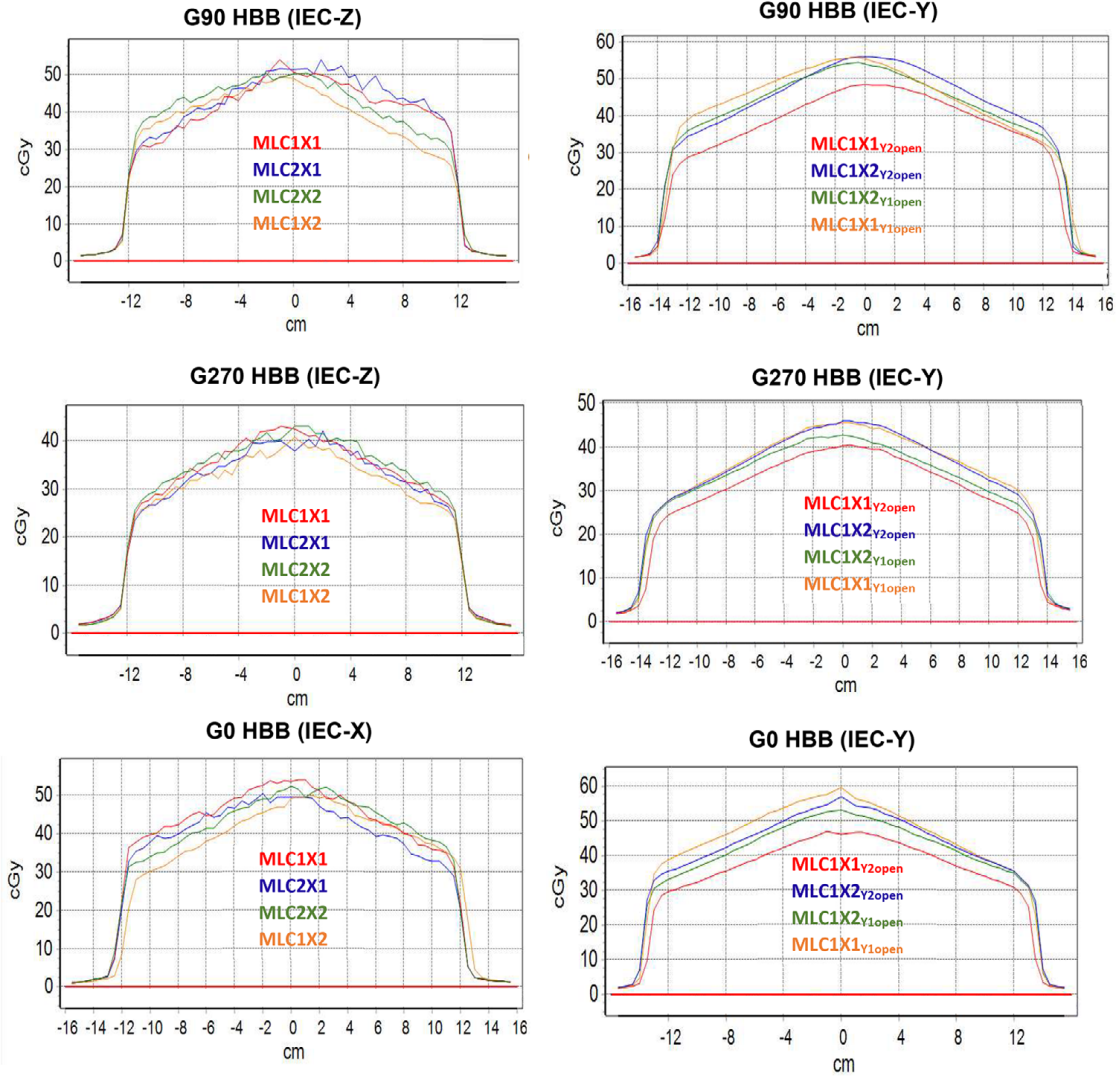
The final IC Profiler (ICP) half beam block (HBB) dose profiles for all observed detector geometries are displayed. IEC‐Y and IEC‐Z were measured at G90 and G270, and IEC‐X and IEC‐Y were measured at G0.

The iterative process of localizing RT isocenter, as measured by the ICP by balancing gantry angle, MLC bank, and couch position, is listed in Tables 5, 6, 7, 8. Specifically, Tables 5, 6, 7, 8 displays the percentage dose difference of the occluded HBB CAX versus open field. Additionally, the CAX dose of the respective HBB is displayed in the Tables 5, 6, 7, 8. The final ICP profiles, after the optimized RT isocenter was determined, are shown in Figure 6. Note that Figure 6 demonstrates that impact of noise observed for the HBB profile defined by the MLC leaf end (i.e., G0 IEC‐X, G90/G270 IEC‐Z) versus the MLC leaf side (i.e., G0 IEC‐Y, G90/G270 IEC‐Y).

**TABLE 5:** Percentage dose difference of half beam block central axis (CAX) versus open field as function of couch displacement to localize radiation isocenter in IEC‐Y.

Couch IEC‐Y position (mm)	0		+0.2		−0.1	
MLC shape/Gantry position	CAX (cGy)	%DD_CAX_	CAX (cGy)	%DD_CAX_	CAX (cGy)	%DD_CAX_
MLC1X1_Y1open_ G90	55.78	55%	52.83	52%	55.16	54%
MLC1X1_Y2open_ G90	47.71	47%	50.97	50%	48.47	48%
MLC1X1_Y1open_ G270	46.14	56%	–	–	40.41	49%
MLC1X1_Y2open_ G270	40.05	49%	–	–	45.91	56%
MLC1X2_Y1open_ G90	56.51	56%	51.73	51%	54.01	53%
MLC1X2_Y2open_ G90	55.63	55%	58.02	57%	56.07	55%
MLC1X2_Y1open_ G270	43.89	54%	–	–	42.68	52%
MLC1X2_Y2open_ G270	45.52	56%	–	–	45.91	56%

**TABLE 6:** Percentage dose difference of half beam block central axis (CAX) versus open field as function of couch displacement to localize radiation isocenter in IEC‐Z.

Couch IEC‐Z position (mm)	0	
MLC shape/Gantry position	CAX (cGy)	%DD_CAX_
MLC1X2 G90	49.02	48%
MLC2X2 G90	50.53	50%
MLC1X2 G270	40.91	50%
MLC2X2 G270	43.27	53%
MLC1X1 G90	50.89	50%
MLC2X1 G90	51.52	51%
MLC1X1 G270	42.48	52%
MLC2X1 G270	37.91	46%

**TABLE 7:** Percentage dose difference of half beam block central axis (CAX) versus open field as function of couch displacement to localize radiation isocenter in IEC‐Y at G0.

Couch IEC‐Y position (mm)	0		+0.7	
MLC shape/Gantry position	CAX (cGy)	%DD_CAX_	CAX (cGy)	%DD_CAX_
MLC1X2_Y1open_ G0	61.89	62%	53.31	53%
MLC1X2_Y2open_ G0	47.74	48%	57.17	57%
MLC1X1_Y1open_ G0	–	–	59.77	60%
MLC1X1_Y2open_ G0	–	–	46.05	46%

**TABLE 8:** Percentage dose difference of half beam block central axis (CAX) versus open field as function of couch displacement to localize radiation isocenter in IEC‐X at G0.

Couch IEC‐X position (mm)	0		−0. 4		−0. 2		−0. 3	
MLC shape/Gantry position	CAX (cGy)	%DD_CAX_	CAX (cGy)	%DD_CAX_	CAX (cGy)	%DD_CAX_	CAX (cGy)	%DD_CAX_
MLC1X2 G0	47.21	47%	48.71	49%	48.71	49%	51.39	51%
MLC2X2 G0	46.13	46%	–	–	–	–	51.38	51%
MLC1X1 G0	58.23	58%	56.34	56%	55.15	55%	53.62	53%
MLC2X1 G0	54.55	54%	–	–	–	–	49.52	49%

The open CAX dose used for profile normalization was 100.3 cGy, 101.8 cGy, and 81.8 cGy for G0, G90 and G270, respectively. Note for simplicity, only the CAX detector was reported in Tables 6, 8 for the profiles defined by MLC leaf ends (i.e., IEC‐Z in G90/G270, and IEC‐X in G0).

#### 3.3.3 G90/G270: IEC‐Z

Table 6 displays the measurements taken to find the IEC‐Z RT isocenter at G90/G270. This %DD was evenly distributed. Therefore, no adjustments were made.

#### 3.3.4 G0: IEC‐Y and G90/G270: IEC‐Y

Table 7 columns 2−3 (IEC‐Y G0) displays the initial measurements taken at the previously determined IEC‐Y G90/G270 position (Table 5). The initial measurements for MLC1 using the X2 bank were deemed to be inconsistent (i.e., 62% vs. 48%). Hence, we shifted the couch +0.7 mm across the IEC‐Y direction. The %DD among the X2 banks after the +0.7 mm shift was more evenly distributed (i.e., 53% vs. 57%). However, the X1 bank then measured a similar %DD discrepancy (60% vs. 46%) as the original couch position, as shown in Table 7.

We decided, despite the discrepancy with the MLC1X1 HBB measurement (60% vs. 46%), to accept this position as the new IEC‐Y isocenter. It was previously determined in the IEC‐Y G90/G270 orientation (Table 5) that the X2 bank was more symmetric in the Leaf 17 and Leaf 18 track (53% vs. 55% at G90, 52% vs. 56% at G270) construction compared to the X1 bank Leaf 17 and Leaf 18 (54% vs. 48% at G90, 49% vs. 56% at G270). This symmetry across X2 bank and asymmetry across X1 bank were observed regardless of couch position (Columns 2−7, Table 5). Therefore, we chose to set IEC‐Y G0 based on the more symmetric X2 bank.

Note that the initial measurements of Table 7 (columns 2−3) showed that HBB Y1 open field created by the X2 bank was overexposed (62%). The X1 bank after the 0.7 mm shift was also overexposed by 60%. However, the X2 bank was *nearly* balanced at this position (53% vs. 57%), based on our previous determination that X2 bank being more symmetrical we favored the results of X2 at this couch position.

If the G0 orientation was further shifted to reduce the X2 bank Y2 overexposure (57%) and better balance the X2 bank result, it would have positioned the detector further toward +IEC‐Y, and hence further from the optimized solution of IEC‐Y found at G90/G270 (Table 5). Keeping in mind that our optimized result was based on X2 bank which we previously had established at G90/G270 as the more symmetric leaf construction pair, we favored X2 balance. The reason why greater %DD was seen at G0 for X1 bank was due to the more sensitive detector orientation across ICP‐Y (i.e., all G0 measurements). Therefore, the final IEC‐Y localization was accepted at this shifted couch position of +0.7 mm, which split the difference between G0 and G90/G270 solution.

#### 3.3.5 G0: IEC‐X

Table 8 displays measurements taken to find the IEC‐X RT isocenter at G0. This %DD was best grouped with couch shift of −0.3 mm.

#### 3.3.6 Lasers to RT isocenter

The lasers are the chain between MR isocenter and RT isocenter as previously described for the MRIdian system.^14^, ^15^ After the final RT isocenter has been localized with the ICP, the lasers were aligned to the detector position. The new laser coordinate was then used to adjust the virtual MR isocenter position in the UI.

#### 3.3.7 Validation of technique: Starshot

Verification of RT isocenter position, as quantified through the magnitude of displacement of the lasers (i.e., pinprick) to radiation CAX, is shown in Table 9 for a starshot and slit test performed on MRIdian. The total magnitude of displacement of the lasers to the radiation CAX was within 0.70 mm.

**TABLE 9:** Verification of radiation (RT) isocenter position as quantified through the magnitude of displacement of lasers to RT central axis (CAX) through film starshot (IEC‐X, IEC‐Z) and film slit (IEC‐Y). The “+ axis” and “‐ axis” represents the position of the respective positive and negative 50% profile for the FWHM analysis.

	IEC‐X (mm)	IEC‐Z (mm)	IEC‐Y (mm)
+ axis	126.2	99.5	94.3
− axis	139.9	108.5	117.4
RT CAX	133.1	104.0	105.8
pinprick	132.4	104.3	105.8
2D displacement CAX to pinprick	0.65	−0.27	0.00
*total magnitude of displacement lasers to RT isocenter (mm)*			0.70

#### 3.3.8 Validation of technique: C‐arm

The difference in the lasers to RT isocenter, as quantified through our ICP RT localization technique to an independent Winston‐Lutz on a C‐arm Linac, was performed at G0 (IEC‐X/IEC‐Y) and G270 (IEC‐Y/IEC‐Z). The respective displacements from lasers were 0.71 mm/0.72 mm (G0) and 0.64 mm/0.29 mm (G270) for ICP/Winston‐Lutz. This demonstrated reproducible results within 0.35 mm between the two techniques.

## 4 DISCUSSION

In the emerging paradigm of stereotactic radiosurgery being proposed for MRgRT, assessment of mechanical geometric accuracy is of upmost importance. In this work, we describe a novel approach to determine RT isocenter location, MLC skew rotation, and beam divergence displacement for an MR Linac system, without requiring an onboard detector or array. We used an HBB technique to irradiate a commercially available MR‐compatible IC array detector with a partially occluded detector. Our technique demonstrated high sensitivity with this real‐time approach. Additionally, this technique is not limited to MRgRT systems; it can be applied broadly on all O‐shaped and C‐arm Linacs. In this work, we validated the accuracy of our HBB approach with a starshot technique using film, and furthermore validated the reproducibility on a conventional C‐arm Linac and compared to Winston‐Lutz.

This proposed method enables instant feedback (real‐time output) to identify RT isocenter compared to a conventional starshot analysis. The starshot measurement is performed on film, and it is recommended that film develop overnight. Additionally, film requires resources for analysis including time of scanning. The ability to take measurements in real‐time allows the user to perform multiple iterations in a single session. This reduces the overall measurement time as it eliminates iterative nature of the starshot measurement. Furthermore, film is a recurring expense, whereas the IC Profiler is a one‐time cost.

This HBB partial detector occlusion technique utilized the partial active volume of the central detector. This enabled a high sensitivity of MLC position with respect to detector position. We quantified this response for both the half beam block with longitudinal and lateral orientation of the ICP detector. We found the sensitivity was 6.4%/mm for the lateral/ICP‐X and 13%/mm for longitudinal/ICP‐Y orientation. Per Equations (1)–(4), the absolute differences observed between the slopes (i.e., %DD_CAX_/(∆_RTisocenter position_) and intercepts are likely due to each system's unique MLC.

Our technique used three of four cardinal gantry angles. This was due to the limitation of shooting through the couch at G180 (i.e., couch attenuation ∼15%). Our previously published technique used an in‐house jig to remove the couch attenuation by positioning the ICP in front of the couch.^10^ For this work, we wanted to create a universal approach without the need for such jig. Note that some sensitivity was reduced due to not utilizing an extended SSD configuration for this work that our previously work demonstrated with the jig (i.e., 6.4%/mm vs. 20.7%/mm). However, for this work we prioritized setting the detector position at true machine isocenter rather than an extended source to detector distance to establish RT isocenter.

Owning to the sensitivity of our method, we were able to detect subtle differences in leaf construction and track path. For example, our results demonstrated leaf differences between the X1 and X2 banks when measuring ICP‐Y orientation as previously discussed in the Results, Tables 5 and 7. As such, during the establishment of the ideal detector position, we found it was more important to balance the measurements with respect to the observed differences between the X1 and X2 banks. However, it is worth noting these differences may not be detectable using other methods (i.e., starshot).

When measuring dose with the ICP‐X geometry, precision is limited by the calibration of each MLC leaf of each respective bank. Small differences were observed among MLC1 and MLC2 (Tables 6 and 8). As previously stated in the Methods to minimize error of any one leaf calibration, a method of balancing across the central ten detectors was used over simply taking the dose difference of the center detector defined by the two central leaves. It is of note in our film validation results that the greatest magnitude of positional difference of lasers to RT isocenter was found across the leaf travel direction (IEC‐X) at 0.65 mm compared to leaf side direction (IEC‐Y) at 0.00 mm. While the film validation still resulted in sub‐mm agreement (i.e., 0.70 mm) of the lasers to RT isocenter position, the larger magnitude of difference is due to reliance of the starshot technique on only two central leaves compared to our ICP technique which allowed balancing across more than two leaves when finding IEC‐X isocenter. Additional uncertainty across IEC‐X may be due to not having a redundant measurement (i.e., IEC‐Y redundant measurement between G90/270 and G0). Moreover, IEC‐X utilizes the ICP‐X geometry which was the less sensitive detector orientation.

A source of error in ICP measurements of RT isocenter could result from the skew between the detector and/or MLC bank. Skew can be quantified by examining the line profile of the HBB taken on the ICP (Figure 1), as previously described in our mechanicals. In this study, we found minimal MLC skew, that is, 0.07 degrees. While an index bar was used to align the ICP array orthogonal on the couch to minimize detector skew for RT isocenter measurements, some detector rotation was noted (Figure 6). This methodology of using the index bar for setup and not correcting for the detector skew for RT isocenter measurements was chosen as it was important to establish a reproducible setup during commissioning for establishing baseline data. Additionally, since we are relying on central detector(s) to quantify RT isocenter, the detector skew (impacting off‐axis detector locations) has a minimal impact on the results.

Additional limitation of this study is that the error of the HBB technique was not measured. Specifically, in this study the MRIdian RT isocenter localization was only performed once on the ICP. One method to assess the uncertainty of the HBB technique is through repeated measurements of the RT isocenter localization with our HBB technique. While an index bar was used in all measurements to minimize dependency on user setup, there still may be user‐variability that would impact the repeatability. Sources of potential ICP setup error include differences in detector positioning along the index bar, which could introduce detector skew related to the isocenter position. Furthermore, since the isocenter is of a finite size, setup uncertainties may be exaggerated with differences in detector alignment/skew relative to isocenter volume. These minor differences in setup can compound into slightly different measurement outcomes. Owning to the sensitivity of the test, such inconsistencies in setup will likely maintain a sub‐mm agreement.

The primary clinical implementation of our proposed technique was to establish the location of RT isocenter with respect to the room lasers during initial commissioning of a ViewRay MRIdian. Once the lasers to RT isocenter have been established, a simpler procedure can be performed for routine QA. For example, we recommend quantifying the displacement between the RT isocenter and lasers on a monthly QA basis through using the CAX position on the ICP for a square field, as we have validated in this work it is reproducible within 1 mm (limitation due to interpolation of detector spacing across FWHM). Additionally, a starshot test could supplement monthly QA to ensure sub‐millimeter accuracy of the mechanical RT isocenter position. Further clinical utilization of this technique includes a major RT mechanical service event such as replacement of Linac or MLC. The proposed technique, once established after commissioning, can then serve as a baseline for comparison for annual QA.

## 5 CONCLUSION

An effective and sensitive method of finding RT isocenter, calculating MLC skew, and measuring beam divergence displacement for an MR Linac has been developed using a commercially available detector array. Our method utilized a variety of detector orientations and gantry angles to measure dose to the central axis of the ICP using HBBs. Each HBB split the central detector in half along the lateral and longitudinal axis such that the dose between the two HBBs were approximately equal. Detector sensitivity measurements were benchmarked to calculate sub‐mm displacements as a function of %DD and then used to calculate RT isocenter, MLC skew, and beam divergence displacement.

Mechanical subtleties such as MLC skew and configuration, which may not be detectable via traditional methods, were able to be quantified, further increasing the precision and accuracy of our technique. This method serves for establishing the foundation of an MR‐guided stereotactic radiosurgery program and achieved the mechanical and geometric precision recommendations of TG 142.

## AUTHOR CONTRIBUTIONS

Nema Bassiri, Ph.D.—data acquisition, writing/editing/revision of manuscript, data analysis. John Bayouth, Ph.D.—conception of study, editing/revision of manuscript. Kathryn Mittauer, Ph.D.—conception of study, data acquisition, writing/editing/revision of manuscript, data analysis

## CONFLICT OF INTEREST STATEMENT

Dr. Bassiri received grant from ViewRay Inc. Dr. Mittauer reports ownership interest in a company that provides consulting services on image guided radiation therapy technology (MR Guidance, LLC.). She received travel reimbursement/consulting fees/speaking fees/grants from ViewRay Inc. Dr. Bayouth reports ownership interest in a company that provides consulting services on image guided radiation therapy technology (MR Guidance, LLC.). He/(his employer) received travel reimbursement/speaking fees from ViewRay Inc.

## Data Availability

Research data are not available at this time.
